# Early results of quality of life for curatively treated rectal cancers in Chinese patients with EORTC QLQ-CR29

**DOI:** 10.1186/1748-717X-6-93

**Published:** 2011-08-12

**Authors:** Junjie Peng, Debing Shi, Karyn A Goodman, David Goldstein, Changchun Xiao, Zuqing Guan, Sanjun Cai

**Affiliations:** 1 Department of Colorectal Surgery, Cancer Hospital Fudan University, Department of Oncology, Shanghai Medical College, Fudan University, Shanghai, China; 2 Department of Radiation Oncology, Memorial Sloan-Kettering Cancer Center, New York, USA; 3 Department of Medical Oncology, Prince of Wales Hospital, Sydney, Australia

## Abstract

**Purpose:**

To assess the quality of life in curatively treated patients with rectal cancer in a prospectively collected cohort.

**Methods:**

Patients with stage I-III rectal cancer who were treated curatively in a single institution were accrued prospectively. Quality of life was assessed by use of the European Organization for Research and Treatment of Cancer questionnaire module for all cancer patients (QLQ-C30) and for colorectal cancer patients (QLQ-CR29). Quality of life among different treatment modalities and between stoma and nonstoma patients was evaluated in all patients.

**Results:**

A total of 154 patients were assessed. The median time of completion for the questionnaires was 10 months after all the treatments. For patients with different treatment modalities, faecal incontinence and diarrhea were significantly higher in radiation group (p = 0.002 and p = 0.001, respectively), and no difference in male or female sexual function was found between radiation group and non-radiation group. For stoma and nonstoma patients, the QLQ-CR29 module found the symptoms of Defaecation and Embarrassment with Bowel Movement were more prominent in stoma patients, while no difference was detected in scales QLQ-C30 module.

**Conclusions:**

Our study provided additional information in evaluating QoL of Chinese rectal cancer patients with currently widely used QoL questionnaires. As a supplement to the QLQ-C30, EORTC QLQ-CR29 is a useful questionnaire in evaluating curatively treated patients with rectal cancer. Bowel dysfunction (diarrhea and faecal incontinence) was still the major problem compromising QoL in patients with either pre- or postoperative chemoradiotherapy.

## Introduction

Colorectal cancer is the second most common cause of cancer death in developed countries and has become an increasingly important health problem in China. Today, multidisciplinary treatment has become the standard strategy in the management of colorectal cancer. In particular, rectal cancer requires a multidisciplinary approach. Patients with transmural disease or node-positive disease may need to receive adjuvant treatment including radiotherapy and/or chemotherapy[1,2]. Although radiotherapy improves local control and disease-free survival, and is favored in most patients with locally advanced disease, the addition of radiotherapy increases toxicity. Chemotherapy can be administered alone for selected cases when patients are not candidates for radiotherapy due to medical conditions, concerns about infertility, or limited access to radiotherapy facilities.

When evaluating the treatment options for rectal cancer patients, consideration of quality of life (QoL) after treatment should be included along with the assessment of survival, local or distant recurrence, treatment morbidity, and toxicity. The European Organization for Research and Treatment of Cancer (EORTC) QoL questionnaire (QLQ) is an integrated system for assessing the health-related QoL of cancer patients. The QLQ-C30 is the core questionnaire for evaluating the QoL of cancer patients. The EORTC QLQ-CR38, the colorectal specific module, was developed originally in the Netherlands and has been widely used in many trials and research settings[3]. The QLQ-CR29 was then developed after revising the QLQ-CR38 for a few years[4], and was demonstrated internationally to have both sufficient validity and reliability to support its use as a supplement to the EORTC QLQ-C30 to assess patient-reported outcomes during treatment for colorectal cancer in clinical trials and other settings[5]. However, the international validation included both patients with rectal and colon cancer. Based on the different treatment modalities and outcome evaluations between colon and rectal cancer, the usefulness of the QLQ-CR29 specifically in rectal cancer needs to be further studied. The primary aim of current study is to assess the QoL in stage I-III rectal cancer with different treatment modalities in a prospectively collected cohort, using QLQ-CR29 as a supplement to QLQ-C30. The impact of a permanent stoma on patients' QoL was also evaluated in our study.

## Patients and Methods

Chinese patients with rectal cancer who were treated with curative intent in the Department of Colorectal Surgery, Fudan University Shanghai Cancer Center between January 2008 and March 2009 were included in the current study. Eligible criteria included 1) age 18-70 years, 3) primary lesion within 12 cm of anal verge, 3) undergoing radical excision of primary lesions, 4) no synchronous distant metastasis, 5) at least 6 months of follow-up after all treatments (including adjuvant treatment), and 6) freedom from local or distant recurrence at the latest follow-up. Since a defunctioning stoma is rarely used in our department[6] and anastomotic leakage may have a negative impact on patients' quality of life[7,8], patients who underwent defunctioning stoma or had postoperative anastomotic leakage were excluded from the study. According to our institutional routine, J-pouch was not used in our series when performing the anastomosis. This study was approved by the ethics committee of the hospital.

Four different treatment regimens were used in this cohort of stage I-III rectal cancer patients: 1) surgery only, 2) surgery plus adjuvant chemotherapy (Surgery+CT), 3) surgery plus adjuvant chemoradiotherapy (Surgery+CRT), and 4) preoperative chemoradiotherapy plus surgery and adjuvant chemotherapy (CRT+Surgery+CT). Questionnaires for QoL were assigned to all of the four groups. Consecutively, each patient who fulfilled the eligibility criteria in our department was asked to participate in the study during their visits for follow-up purposes. Each group was designed to accrue a total of 40 patients. Specifically, for female patients, menopausal status was recorded and none of the patients received hormone-replacement treatment.

### Treatment Protocol and Follow-up

All patients in the project had preoperative staging by rectal magnetic resonance imaging. Preoperative T4 or node-positive patients were referred for preoperative chemoradiotherapy. Preoperative T3 patients were recommended to receive either chemoradiotherapy before surgery or surgery first. Preoperative T1-2 patients and patients who were unwilling or unable to have radiotherapy also underwent surgery first. For patients whose tumors were located above the peritoneal reflection or over 10 cm from anal verge, surgery was generally performed first. All patients who received preoperative chemoradiotherapy were planned to receive 4-6 cycles of fluorouracil-based chemotherapy; pT4N0 or pTanyN1-2 patients were planned to receive adjuvant chemoradiotherapy or adjuvant chemotherapy for those unwilling or unable to have radiotherapy; pT1-2N0 patients did not have any adjuvant treatment; pT3N0 patients were asked to choose the protocol similar to pT4N0 or pT1-2N0 at the discretion of the treating physician.

According to the institutional protocol, each patient was asked to return for follow-up every 3 months after the radical excision of primary tumor for the first 3 years. During follow-up, each eligible patient was asked to participate in the study and informed consent was signed. Each patient was asked to finish the questionnaires at the hospital and a research nurse was present to help if needed.

### Measures and Analyses

The EORTC QLQ-C30 (version 3.0) and QLQ-CR29 questionnaires are used in current study. The QLQ-C30 is composed of both multi-item scales and single-item measures. These include five functional scales, three symptom scales, a global health status, and six single items[9]. The QLQ-CR29 is meant for use among colorectal cancer patients varying in disease stage and treatment modality. The module comprises 29 questions assessing the colorectal cancer-specific symptom scales (disease symptoms, side effects of treatment) and functional scales (body image, sexuality, and future perspective) [4]. All scales and single-items measures in both questionnaires are linearly transformed to give a score from 0 to 100 according to the algorithm recommended by developers. A high score for a functional scale represents a high level of functioning, a high score for the global health status represents a high QoL, and a high score for a symptom scale represents a high level of symptomatology or problems. For items without a response, at least 75% of items completed by patients are considered assessable in the current study, and the mean was imputed for missing items in assessable cases according to EORTC scoring guidelines[10].

### Statistics

The distribution of the demographic and clinical characteristics was tested by one-way analysis of variance (ANOVA) for continuous variables and chi-square test for classified variables. Nonparametric test was used to compare the differences of the scales/items in QLQ-C30 and QLQ-CR29. For comparing the QoL in stoma and nonstoma patients, differences were obtained by comparing the distribution between groups (stoma and nonstoma), using the Mann-Whitney U test for 2 samples. This method was also used for comparing the QoL in patients with or without radiotherapy. For comparing the QoL in patients with four different treatment regimens, Kruskal-Wallis one-way ANOVA for *k *samples was used and Bonferroni correction was used in multiple comparisons. A p < 0.05 was considered statistically significant.

## Results

### Patients

A total of 182 patients were identified who met the criteria of a minimum of 6 months since completion of all treatment and were asked to participate in the study, of whom 23 patients declined and 5 patients' questionnaires were not evaluable due to too many missing items. We now report upon the first round of tumor assessment and the patient reported outcomes in different treatment groups. One hundred fifty four questionnaires (84.6%), including both the QLQ-C30 and CR29, were assessable and enrolled in the current study. The mean time of completion for the questionnaires was 9.8 months (range, 6-15 months) after all the treatments. The mean time from end of treatment to QoL assessment was similar among groups with different treatments and between stoma and nonstoma group. The baseline characteristics of the four groups with different treatment modalities are listed in Table 1 together with the mean time of questionnaire administration. In all the 154, only 3 patients (2%) were unmarried or divorced. The mean age in the Surgery Only and CRT+Surgery+CT groups was higher than that of Surgery+CT and Surgery+CRT (p = 0.022). The mean distance from anal verge of the primary tumor was 5.4 cm in groups with radiotherapy and 7.0 cm in groups without radiotherapy. Multicomparison found that patients in the CRT+Surgery+CT group had significantly lower disease than in the other three groups, which had similar location of disease compared with each other. Most of pTNM stage I (75.7%) patients underwent surgery only, while three patients received adjuvant chemotherapy due to neural or vascular invasion; the other six patients received neoadjuvant chemoradiotherapy and postoperative pTNM turned out to be pTNM stage I disease.

**Table 1:** Baseline characteristics of the four treatment modality groups

		Total n = 154 (100%)	Surgery Only n = 34 (22%)	Surgery+CT n = 40 (26%)	Surgery+CRT n = 40 (26%)	CRT+Surgery+CT n = 40 (26%)	P Value
Gender (%)	Male	88 (57.1)	18 (11.7)	24 (15.6)	21 (13.6)	25 (16.2)	0.753
	Female	66 (42.9)	16 (10.3)	16 (10.4)	19 (12.4)	15 (9.8)	
Mean time to assessment, months (*SD*)*		10.4 (2.9)	9.8 (2.5)	9.6 (2.2)	9.7 (2.5)	9.7 (2.2)	0.879
Median age (range)		57 (30-70)	54 (26-70)	52.5 (27-68)	52 (26-70)	55.5 (39-69)	0.004
Mean distance from anal verge, cm (range, *SD*)		6.9 (2-12, 2.9)	6.2 (1-12, 2.9)	7.0 (1-12, 2.7)	5.9 (1-12, 3.2)	4.8 (1-12, 2.2)	0.002
Postoperative pTNM stage (%)	Stage I	37 (24)	28 (18.2)	3 (1.9)	0	6 (3.9)	<0.001
	Stage II	44 (28.6)	6 (3.1)	18 (11.7)	11 (7.1)	9 (5.8)	
	Stage III	73 (47.4)	0	19 (12.4)	29 (18.9)	25 (16.3)	
Stoma (%)	Yes	75 (48.7)	14 (9.1)	14 (9.1)	22 (14.3)	25 (16.2)	0.06
	No	79 (51.3)	20 (12.9)	26 (16.9)	18 (11.7)	15 (9.8)	

CRT, chemoradiotherapy; CT, chemotherapy; SD, Standard Deviation

* Time to assessment refers to the time from end of all treatment to QoL assessment

The relationships between age and the functional or symptomatic scales were studied by correlation analyses. In functioning scales, patient age was found positively correlated with the Body Image scale (p = 0.029, correlation coefficient 0.18) and negatively correlated with the Female Sexual Function scale (p = 0.0002, correlation coefficient -0.44). In symptom scales, age was found negatively correlated with the Embarrassment With Bowel Movement scale (p = 0.007, correlation coefficient -0.221) and dyspareunia (p = 0.002, correlation coefficient -0.386).

### QOL Among Patients with Different Treatment Protocols

QoL was also compared among groups of patients with different treatment modalities (Table 2). In QLQ-C30, no difference was found for functional scales/items among the four groups, while diarrhea in the symptom scale was found significantly differently distributed among the four groups. In QLQ-CR29, no functional scales/items were found to be different between the four groups, while in symptom scales/items, the Faecal Incontinence and Bloated Feeling scales were significantly different among groups (p = 0.006 and 0.003, respectively).

**Table 2:** Quality of life for different treatment modalities

	Surgery Only (n = 34)		Surgery+CT (n = 40)		Surgery+CRT (n = 40)		CRT+Surgery+CT (n = 40)		P Value
	Mean	Median (range)	Mean	Median (range)	Mean	Median (range)	Mean	Median (range)	
Globe health status	67	75 (25-83)	64	75 (25-83)	69	75 (25-92)	62	75 (25-92)	0.464
*Functional Scales/Items*									
*EORTC QLQ-C30*									
Physical functioning	64	67 (20-93)	67	73 (13-93)	69	73 (13-93)	70	73 (40-93)	0.529
Role functioning	59	67 (17-100)	59	67 (17-100)	55	50 (0-100)	60	67 (0-100)	0.496
Emotional functioning	55	58 (17-100)	54	58 (25-100)	56	58 (25-100)	58	58 (17-100)	0.774
Cognitive functioning	68	83 (33-100)	69	83 (17-100)	71	83 (33-100)	72	83 (33-100)	0.814
Social functioning	72	83 (0-100)	66	75 (0-100)	73	83 (17-100)	71	83 (0-100)	0.563
*EORTC QLQ-CR29*									
AAnxiety	84	83 (50-100)	87	83 (50-100)	86	83 (50-100)	88	83 (50-100)	0.145
Body image	96	100 (67-100)	98	100 (67-100)	95	100 (44-100)	99	100 (78-100)	0.685
Male Sexual function	61	67 (33-100)	60	67 (0-100)	57	67 (0-100)	64	67 (0-100)	0.968
Female Sexual function	48	50 (0-100)	48	33 (0-100)	53	33 (33-100)	42	33(0-100)	0.529
									
*Symptom Scales/Items*									
*EORTC QLQ-C30*									
Fatigue	38	33 (22-67)	43	44 (22-100)	39	33 (0-89)	35	33 (0-78)	0.409
Nausea and vomiting	5	0 (0-33)	3	0 (0-33)	6	0 (0-33)	3	0 (0-17)	0.208
Pain	18	17 (0-67)	21	17 (0-67)	20	17 (0-50)	16	17 (0-50)	0.627
Dyspareunia	5	0 (0-67)	12	0 (0-67)	10	0 (0-33)	5	0 (0-33)	0.124
Insomnia	30	33 (0-100)	21	0 (0-100)	22	17 (0-100)	31	33 (0-100)	0.158
Appetite loss	10	0 (0-67)	18	0 (0-67)	14	0 (0-67)	12	0 (0-67)	0.375
Constipation	24	33 (0-100)	23	33 (0-67)	20	33 (0-67)	21	33 (0-67)	0.923
Diarrhoea	26	33 (0-67)	32	33 (0-67)	45	33 (0-100)	48	33 (0-100)	0.001
Financial difficulties	60	67 (0-100)	59	67 (0-100)	68	67 (0-100)	63	67 (0-100)	0.509
*EORTC QLQ-CR29*									
Micturition problems	8	0 (0-67)	9	0 (0-56)	6	0 (0-33)	6	0 (0-33)	0.844
Abdominal and pelvic pain scale	7	0 (0-22)	7	0 (0-33)	8	0 (0-33)	5	0 (0-44)	0.396
Defaecation problems	10	8 (0-33)	9	8 (0-42)	10	8 (0-25)	9	0 (0-75)	0.321
Faecal incontinence scale	19	17 (0-27)	23	17 (0-67)	28	33 (0-50)	30	33 (0-67)	0.006
Bloated feeling	5	0 (0-67)	9	0 (0-67)	14	0 (0-67)	3	0 (0-33)	0.003
Dry mouth	6	0 (0-33)	7	0 (0-33)	5	0 (0-33)	17	0 (0-67)	0.071
Hair loss	2	0 (0-33)	9	0 (0-100)	7	0 (0-67)	7	0 (0-100)	0.422
Trouble with taste	4	0 (0-67)	3	0 (0-67)	7	0 (0-33)	2	0 (0-33)	0.072
Sore skin	7	0 (0-67)	8	0 (0-33)	10	0 (0-67)	9	0 (0-67)	0.815
Embarrassed by Bowel Movement	9	0 (0-67)	8	0 (0-33)	10	0 (0-33)	6	0 (0-33)	0.572
Stoma related problems	5	0 (0-33)	7	0 (0-33)	16	0 (0-33)	11	0 (0-67)	0.161
Impotence	13	0 (0-67)	15	0 (0-67)	19	0 (0-100)	32	33 (0-100)	0.215
Dyspareunia*	0	0 (0-0)	5	0 (0-33)	21	0 (0-100)	0	0 (0-0)	0.004

* this item was analyzed in female patients only

To evaluate if the differences in functional/symptom results were caused by the addition of radiation, the Surgery+CRT group and CRT+Surgery+CT group were combined as radiation group, the Surgery only group and Surgery+CT group were combined as non-radiation group. Nonparametric test revealed that faecal incontinence and diarrhea were significantly higher in radiation group (p = 0.002 and p = 0.001, respectively). Bloated Feeling was not found different between the two groups.

In the sexual function scales/items, only Dyspareunia was found differently distributed among the four groups (p = 0.004). However, no difference in male or female sexual function was found between radiation group and non-radiation group.

### Quality of Life Between Stoma and Nonstoma Patients

More male patients were found to have a permanent stoma than female patients (33.1% in male *vs *15.6% in female, *p *= 0.008), and stoma patients had lower primary disease than nonstoma patients (mean distance from anal verge: 4.1 cm in stoma patients *vs *8.1 cm in nonstoma patients, *p *= 0.002). Other characteristics, including age, tumor stage, postoperative treatment, are similar between two groups. Of all the 154 patients, the QLQ-C30 questionnaire failed to detect any differences in general QoL between stoma and nonstoma patients (Table 3). However, the colorectal module QLQ-CR29 found that the symptom of Defaecation was more common in nonstoma patients (p = 0.005), while Embarrassment With Bowel Movement were more prominent in stoma patients (p = 0.00001). Although the score of Body Image in our series was high in both of the two groups, the nonstoma patients were more satisfied with their body images (p = 0.031). 92% of nonstoma patients had a score of 100, while 80.8% of stoma patients had a score of 100. Other functional or symptom scales in QLQ-CR29 were not found to be significantly different between stoma and nonstoma patients.

**Table 3:** Quality of life for stoma and nonstoma patients

	Stoma (n = 75)		Nonstoma (n = 79)		P Value
	Mean	Median (range)	Mean	Median (range)	
Globe health status	66	75 (25-92)	64	75 (25-92)	0.183
*Functional Scales/Items*					
*EORTC QLQ-C30*					
Physical functioning	68	73 (13-93)	67	73 (20-87)	0.889
Role functioning	57	67 (0-100)	59	67 (0-100)	0.969
Emotional functioning	55	58 (25-100)	57	58 (17-100)	0.301
Cognitive functioning	72	83 (33-100)	69	83 (17-100)	0.143
Social functioning	67	67 (0-100)	74	83 (0-100)	0.698
*EORTC QLQ-CR29*					
Anxiety	87	83 (50-100)	86	83 (50-100)	0.923
Body image	95	100 (44-100)	98	100 (67-100)	0.031
Male sexual function	60	67 (0-100)	61	67 (0-100)	0.917
Female sexual function	40	33 (0-100)	36	33 (0-100)	0.785
					
*Symptom Scales/Items*					
*EORTC QLQ-C30*					
Fatigue	40	33 (22-100)	37	33 (0-100)	0.268
Nausea and vomiting	4.7	0 (0-33)	6	0 (0-33)	0.588
Pain	17	17 (0-67)	20	17 (0-67)	0.391
Dysponea	9	0 (0-67)	7	0 (0-67)	0.204
Insomnia	25	33 (0-100)	26	33 (0-100)	0.668
Appetite loss	13	0 (0-67)	14	0 (0-67)	0.652
Constipation	21	33 (0-100)	22	33 (0-67)	0.793
Diarrhoea	41	33 (0-100)	35	33 (0-67)	0.334
Financial difficulties	64	67 (0-100)	61	67 (0-100)	0.459
EORTC QLQ-CR29					
Micturition problems	6	0 (0-44)	9	0 (0-67)	0.639
Abdominal and pelvic pain scale	7	0 (0-33)	6	0 (0-44)	0.363
Defaecation problems	7	0 (0-33)	12	8 (0-75)	0.005
Faecal incontinence scale	25	17 (0-67)	25	17 (0-67)	0.871
Bloated feeling	8	0 (0-67)	7	0 (0-67)	0.916
Dry mouth	8	0 (0-67)	9	0 (0-67)	0.416
Hair loss	6	0 (0-100)	6	0 (0-100)	0.921
Trouble with taste	3	0 (0-67)	4	0 (0-67)	0.938
Sore skin	10	0 (0-67)	7	0 (0-67)	0.105
Embarrassed by bowel movement	14	0 (0-67)	3	0 (0-33)	0.00001
Stoma related problems	11	0 (0-67)	-	-	-
Impotence	25	0 (0-100)	19	0 (0-100)	0.242
Dyspareunia*	13	0 (0-100)	4	0 (0-67)	0.173

* this item was analyzed in female patients only.

## Discussion

This study examined the additional benefit of using the QLQ-CR29 as a supplement to the QLQ-C30 in patients with rectal cancer treated with different treatment protocols. Our study was conducted in a prospectively collected series of patients, and each patient was asked to complete the questionnaires at the time of the follow-up visit. The proportion of patients completing the questionnaires was 84.6%, which was similar to previous studies[5]. To our knowledge, this is the first study focused on the QoL of treated patients with rectal cancer using the QLQ-CR29. Our study demonstrated that the QLQ-CR29 was able to provide additional information about patient outcomes in almost all kinds of rectal cancer patients who were curative treated. We also assessed the utility of the questionnaires in identifying differences in stoma and nonstoma patients. Since the meantime to QoL assessment were similar across all our groups our findings are unlikely to be due to differences in timing of the assessments. Similarly ensured that assessments only began after 6 months had elapsed from completion of all therapy. Previous studies have shown that most patient reported outcomes tend to have improved or stabilized by that time point[11]. Our data only addresses the early impact at the end of the first year following treatment. Additional follow-up will be required to look for late effects of treatment on patients' QoL.

Local recurrence is one of the major problems in the treatment of rectal cancer. Radiotherapy or chemoradiotherapy was introduced into this field due to the reduction of local recurrence for locally advanced rectal cancer[1,12]. However, the toxicity of radiotherapy has been criticized and the long-term results of the toxicity among different treatment regimens are seldom studied. Bowel functions, urinary incontinence, and sexual functions are the most-reported complaints that may affect the use of radiotherapy. Otherwise, infertility considerations and convenience to the facility of radiotherapy are other reasons that may reduce the use of radiotherapy for patients. We found that the responses of patients to the QLQ-C30 were broadly similar to previous studies[11,13,14]. Marijnen et al. found short-term preoperative radiotherapy resulted in more sexual dysfunction, slower recovery of bowel function, and impaired daily activity postoperatively[15]. Pucciarelli et al ever reported that patients with preoperative radiotherapy had worse outcomes for bowel function, including constipation, diarrhea, stool fractionation, use of enema/laxative, urgency, and sensation of incomplete evacuation[16]. But these impaired functions were compared with the general population, so the surgery-related issues could not be balanced, and the time spectrum of completing the questionnaires was not provided. In our series, a higher rate of diarrhea and faecal incontinence was also observed in patients with radiotherapy. However, the patients who received pre- or postoperative chemoradiation had more distal tumors, in which cases the surgery would have required a very low anastomosis and therefore may have resulted in worse sphincter function.

Sexual functions and symptoms are the most difficult scales from which to draw conclusions, as many patients are reluctant to complete the questions or give the truth to doctors. Some studies were unable to evaluate sexual functions due to too many missing values. Previous studies found total mesorectal excision or preoperative short-tem radiotherapy had a negative effect on sexual functioning in males and females[15]. Although we found the symptom of dyspareunia was higher in patients with postoperative chemoradiotherapy, none of male or female sexual function was found significantly different between radiation group and non-radiation group. One explanation may be that patient with postoperative chemoradiotherapy have shorter times to recover from the radiotherapy, compared with patients whose radiotherapy were delivered preoperatively. Meanwhile, we also found that patient age was correlated to female sexual function and dyspareunia. Multivariate analysis wasn't reported in analyzing the impact of sexual function in patients with or without radiotherapy, so the interaction between demographic features and clinical features in patients with multimodality treatment is still unknown to us.

The extent of the difference between stoma and nonstoma patients in quality of life remain controversial, and have been tested in a variety of studies, mainly based on QLQ-C30 and CR38. Early studies found stoma patients suffered higher levels of psychologic distress and had more problems in social functioning, as well as the sexual functions[17]. However, recently other studies found that the QoL in stoma patients was not inferior to nonstoma patients, and even better in some functional scales. Krouse et al found that both male and female cases with stoma had significantly worse social well-being compared with nonstoma cases, while only female cases reported significantly worse overall health-related QoL and psychological well-being[18]. A meta-analysis reported by Cornish et al. revealed that no difference was found in globe health scores between the two groups[19], although stoma patients were inferior in physical function and sexual function, while the cognitive and emotional functions in stoma patients were superior to nonstoma patients. Other studies also found nonstoma patients had more gastrointestinal complaints, diarrhea, and constipation, and even had lower scores in global health status and future perspective[14,15,20]. In our study, as was expected; the embarrassment with bowel movement symptom of was more common in stoma patients. However, defaecation problem was more prominent in nonstoma patients. The existence of an anastomosis and surrounding chronic inflammation may attribute to this symptom.

Another impaired function scale found in stoma patients was the body image scale. Although the mean and median values of body image were similar between stoma and nonstoma patients, the distribution was significantly different between the two groups: 92.4% in nonstoma patients scored 100 in the body image function scale, compared with 80.8% in stoma patients. Similar results of undermining body image due to a permanent stoma were also reported in previous studies[13,21-23]. However, the score in body image seems higher than the score in the published literature based on Caucasians[13,20], and similar high scores were also observed in studies including patients in Hong Kong and Taiwan[24]. Cultural differences and less obese populations may account for these disparate findings. Another possible reason may be that in our study, 98% of patients are married while less than 80% of married patients were reported in previous studies[5,11,14]. Similar to several recent studies[14,25], no significantly difference was found for male and female sexual function and sexual related symptoms in our study.

However, as the current study mainly focused on the differences of quality of life among different treatment groups, a longitude assessment of QoL before and after treatment was not conducted for each patient. The relationship between the impaired functional results and preoperative status of individuals is unknown to us. Further study is needed to clarify this issue. Since quality of life is a relatively subjective variable, differences in human race, culture, education, religion and social environment, will have impacts on the results. International cooperation is needed to study the quality of life in patients with multiple cultural backgrounds.

## Conclusions

Our study provided additional information in evaluating QoL of Chinese rectal cancer patients with currently widely used QoL questionnaires. By using the EORTC QLQ-CR29 as a supplement to the QLQ-C30, we assessed the QoL in rectal cancer patients with different treatment regimens, as well as the impact of a permanent stoma on patients' QoL. Bowel symptoms (diarrhea and faecal incontinence) were still significant in patients with either pre- or postoperative chemoradiotherapy, and similar QoL was also observed in stoma and nonstoma patients. Additional follow-up will be required to look for late effects of treatment on patients' QoL.

## Conflict of interests statement

The authors declare that they have no competing interests.

## Authors' contributions

JP and DS designed the study, analysis and interpretation of the data, and drafted the article. DG participated the study design and revised the manuscript. KG revised the manuscript and provided important intellectual content. CX participated in the acquisition and analysis of data. ZG participated in interpretation of data and revision of manuscript. SC participated the study design, interpreting the data, and responsible for final approval of the manuscript. All authors have read and approved the final manuscript.
